# Retrocaval ureter and right ureteropelvic junction obstruction: a case report and literature review

**DOI:** 10.3389/fmed.2025.1520235

**Published:** 2025-02-21

**Authors:** Ze-Sheng Jiang, Min-Bo Yan, Zhuan Lv, Ji-Rong He, Wei-Jun Wen, Chuan-Xin Mai, Fa-Rong Tu, Jian-Sheng Li

**Affiliations:** ^1^Department of Urology, Heshan People's Hospital, Jiangmen, China; ^2^Department of Urology, Fifth Affiliated Hospital of Sun Yat-sen University, Zhuhai, China

**Keywords:** retrocaval ureter, ureteropelvic junction obstruction, renal artery variation, hydronephrosis, laparoscopic surgery

## Abstract

A clinical case involving a patient with retrocaval ureter and right ureteropelvic junction obstruction (UPJO) is presented, accompanied by a comprehensive review and discussion of relevant literature. The patient, a 43-year-old female, was admitted to the hospital after discovering right hydronephrosis 2 weeks prior. Computed tomographic urography (CTU) revealed significant right hydronephrosis, a retrocaval ureter, and compression of the right renal variant artery causing UPJO. Retrograde pyelography further demonstrated a stenotic upper segment of the right ureter, exhibiting an “S”-shaped appearance. To address these issues, the patient underwent laparoscopic surgery for retrocaval ureteral realignment and right pyeloureteroplasty. Notably, there were no complications during or after the surgical procedure, and the patient’s recovery was uneventful. The coexistence of retrocaval ureter and right UPJO is infrequently encountered in clinical practice. However, the simultaneous correction of these anomalies through laparoscopic surgery has proven to be both safe and feasible.

## Introduction

The occurrence of variations in the inferior vena cava (IVC) is uncommon in clinical practice (1), similarly, renal artery anomalies are also rare, and the coexistence of both anomalies is exceptionally unusual. The majority of patients with such anomalies display no discernible clinical symptoms or signs. Presently, the extensive utilization of imaging modalities, such as computed tomography (CT), facilitates precise preoperative diagnosis and minimizes the risk of intraoperative complications (2). In April 2024, our department managed a case involving a patient with a retrocaval ureter and right ureteropelvic junction obstruction. A detailed report of the case, accompanied by a review of pertinent literature, is presented below.

### Medical history

A 43-year-old female patient was admitted to our department on April 21, 2024, after discovering right hydronephrosis 2 weeks earlier. Two weeks prior, the patient underwent a color Doppler ultrasound examination specifically for thyroid nodule disease. Given concerns about potential health issues in other bodily regions, supplementary ultrasound scans were also conducted on the urinary system. The results of the urinary tract ultrasound revealed a hydronephrosis measuring approximately 32 mm and a parenchyma thickness of roughly 18 mm. The patient indicated that they did not exhibit any discernible clinical symptoms. Physical examination revealed no abdominal masses, and percussion tenderness was absent in both kidneys. She was subsequently admitted for further diagnostic evaluation and treatment.

Preoperative laboratory tests showed a serum creatinine level of 66 μmol/L and an estimated glomerular filtration rate of 98.7 mL/min/1.73m^2^.

### Imaging findings

#### CTU

**Figure 1:**
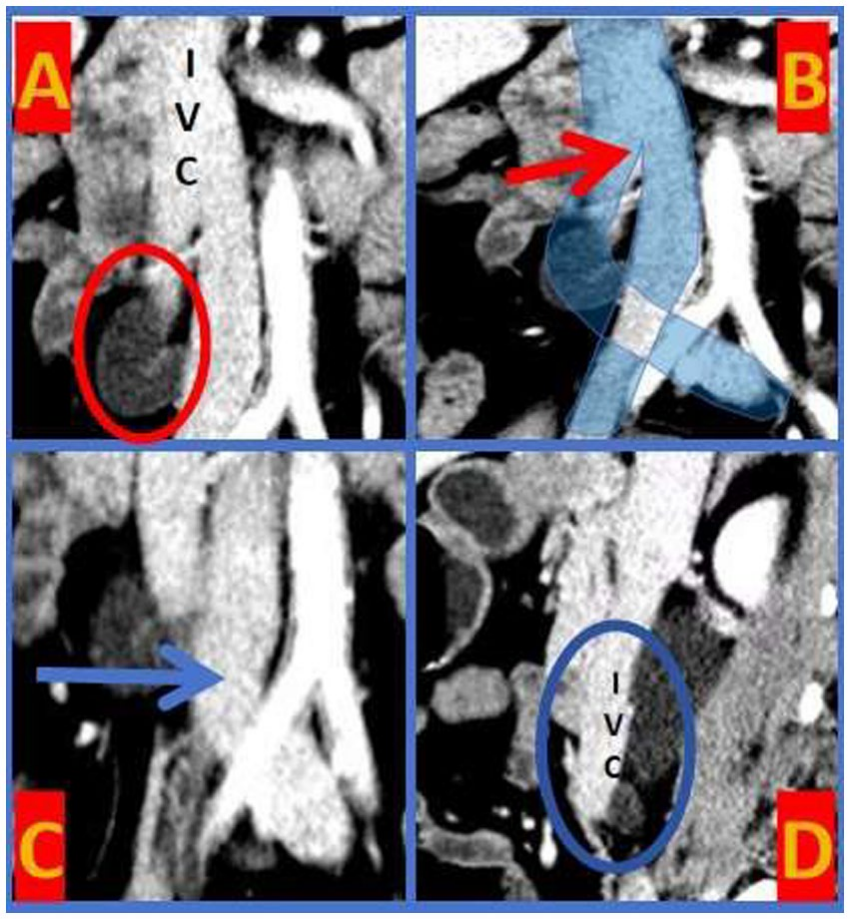
IVC Bifurcation Malformation. **A**-circle: the upper segment of the right ureter exhibited a “fishhook-shaped” appearance. **B**-arrow: the IVC bifurcated at the level of the first lumbar vertebra (L1). **C**-arrow: the bifurcations of the IVC crossed at the level of the third lumbar vertebra (L3). **D**-circle: the tortuous segment of the right ureter was positioned posterior to the IVC.

**Figure 2:**
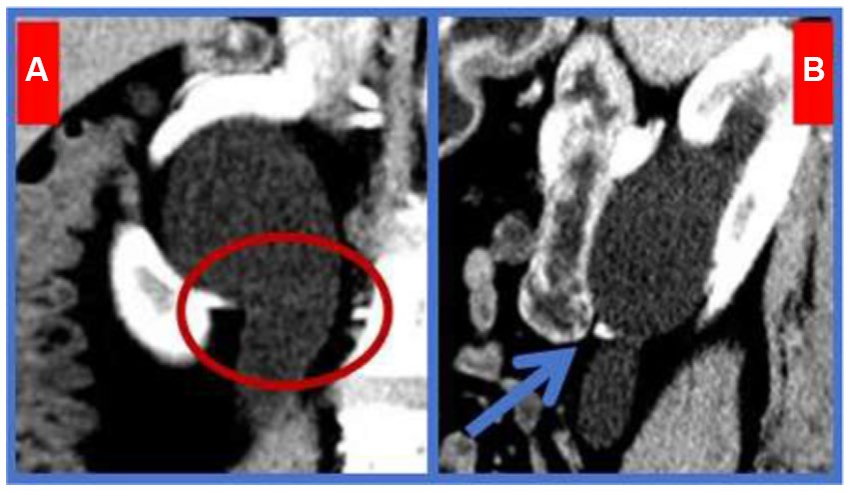
Right Ureteropelvic Junction Obstruction. **A**-circle: A variant artery originating from the lower pole of the right kidney traversed the pyeloureteral junction. **B**-arrow: The variant artery was located ventral to the junction, causing significant depression and stenosis.

The IVC bifurcated at the L1 level and crosses at L3 to form the left and right common iliac veins (Figure 1). The right kidney and the upper segment of the right ureter exhibited dilated effusion. The upper segment of the right ureter, shaped like a fishhook, was located posterior to the IVC. A branch artery of the abdominal aorta crossed the right inferior pole of the kidney (Figure 2), traversing the ventral side of the right pyeloureteral junction, where a notable depression and stenosis were observed.

#### Retrograde pyelography

**Figure 3:**
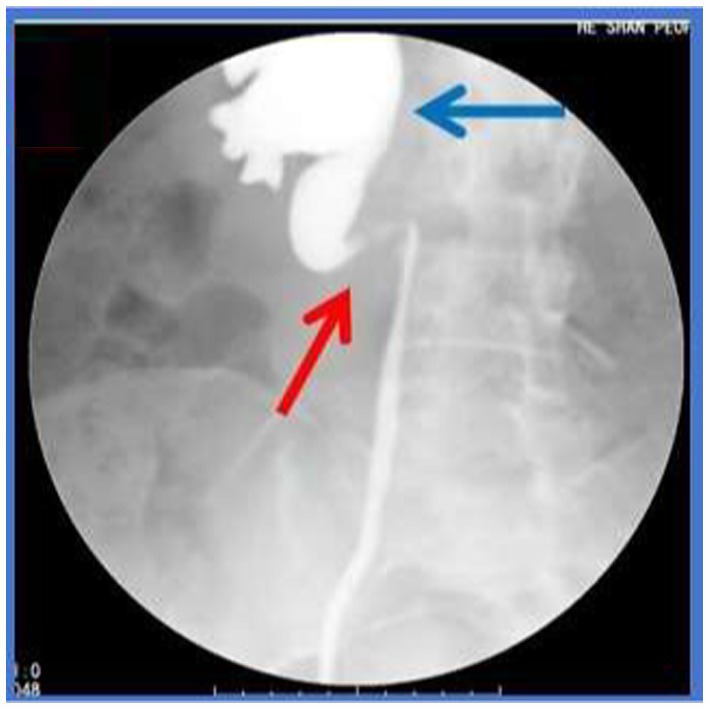
Retrocaval Ureter Deformity (Retrograde pyelography). Red arrow: the upper segment of the right ureter assumed an “S”-shaped configuration. Blue arrow: the right renal pelvis was dilated.

Injection of contrast agent through the urethral catheter revealed dilation of the right renal pelvis and an “S”-shaped upper segment of the right ureter (Figure 3). The narrow segment measured approximately 23 mm in length.

### Surgical procedure

**Figure 4:**
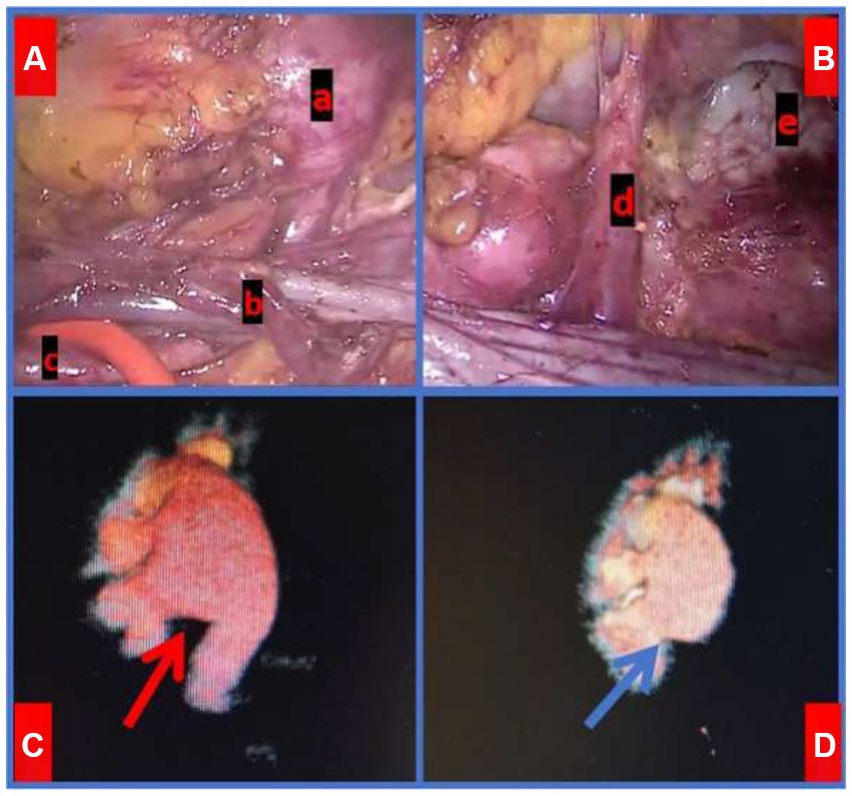
Intra-op laparoscopic findings and comparison of Imaging Pre- and Post-Surgery. **A**-a: Significantly dilated ureter. **A**-b: the right ureter was positioned posterior to the IVC. **A**-c: NORMAL ureter below the obstruction. **B**-d: the variant artery located ventral to the UPJ. **B**-e: Right renal pelvis. **C**-red arrow: Preoperative CTU revealed dilation of the right renal pelvis. **D**-blue arrow: CTU demonstrated right renal pelvis dilation 3 months post-surgery.

After excluding contraindications, including severe cardiopulmonary insufficiency, bleeding disorders or coagulation deficiencies, critical intra-abdominal infections, and the presence of a solitary kidney, among various others, the patient underwent surgery on April 23, 2024, consisting of a classic laparoscopic retrocaval ureteral realignment and right pyeloureteroplasty. Intraoperatively, the altered IVC was identified at the subrenal pole level, dividing into left and right branches. The right ureter was redirected below the IVC bifurcation to the posterior aspect of the right IVC branch. A fishhook-like deformation was noted in the compressed ureter segment, with a diameter of approximately 1.5 cm above the compression. A transverse ectopic artery (approximately 0.3 cm in diameter) compressed the ureteropelvic junction at the kidney’s lower pole, causing significant renal pelvis dilation above the compression site (Figure 4). The stenosed ureter segment was resected, and the proximal ureter was freed from behind the IVC. The compressed segment was severely narrowed, nearly atretic, with no observable urine outflow. The narrowed ureter segment was completely resected, and the ureteropelvic junction was bypassed to position the variant artery dorsally relative to the ureter. The ureteral discontinuity was repaired using continuous suturing with vicryl suture, and the surgical incision was closed.

### Postoperative outcome

Three months post-surgery, the patient returned for a follow-up visit with no apparent clinical symptoms or signs. Her serum creatinine level was 52 μmol/L, and the estimated glomerular filtration rate improved to 112.8 mL/min/1.73m^2^.

## Discussion

IVC, the largest vein in the human body, typically forms by the confluence of the left and right common iliac veins at the level of the fifth lumbar vertebra, with some variations converging at the fourth lumbar level. Variations in the IVC can generally be categorized into six types (1), including the absence of the hepatic segment, compensatory drainage of the superior vena cava via the azygos or hemiazygos veins; translocation of the IVC; double IVC malformation; ureter surrounding the IVC (retrocaval ureter); circumaortic left renal vein; and posterior aortic left renal vein. The retrocaval ureter, specifically, arises due to the development of the inferior renal vena cava from the right posterior main vein, causing the right ureter to traverse behind the IVC and descend to the right side of the abdominal aorta. This anomaly occurs in approximately 0.13% of the population (3). Although congenital, most patients remain asymptomatic (4) and are usually diagnosed between ages 30 and 40 (5). Bateson et al. (6) classified the retrocaval ureter into two types: Type I, the most common, features a fishhook, S-shape, or inverse J-shape configuration; and Type II, characterized by a sickle-shape with the upper ureter shifting to the midline at the pelvic level. In this case, CTU and Retrograde pyelography revealed typical fishhook-like changes in the ureter, confirming a Type I retrocaval ureter. Notably, the patient exhibited an uncommon presentation combined with IVC bifurcation malformation, where the IVC divided into left and right branches at L1 with the branches crossing at L3 (Figure 1).

UPJO refers to the obstruction of urine flow from the renal pelvis to the ureter, potentially leading to progressive kidney damage if undetected and untreated (7). UPJO affects approximately 0.05 to 0.13% of the population (8). Common causes include intraluminal ureteral stenosis, polyps, and valves; high ureteropelvic junction; dynamic dysfunction of the ureteropelvic junction; fibrous cord compression; and pressure from renal artery variations, which account for about 45% of cases (9). Renal artery variations can be categorized into two types: (1) Anterior renal artery branches, where those originating from the renal artery within 15 mm of its root are considered early branches (10); (2) Accessory renal arteries, where one kidney has more than one additional artery originating from the abdominal aorta or its branches (11). Reports indicate that accessory renal arteries occur in about 25.9% of cases, with those running on the ventral side of the pyeloureteral junction and directly into the inferior pole of the kidney occurring in about 6.3% (12), supplying 20 to 25% of renal parenchyma blood (13). The patient’s CTU showed that the right renal variant artery was an accessory renal artery originating from the abdominal aorta and merging transversely into the lower pole of the right kidney. This artery crossed the ventral side of the right ureteropelvic junction, causing compression and subsequent renal pelvis dilation (Figure 4).

Treatment for UPJO caused by ectopic blood vessels often requires tailored approaches based on the vessel’s location and direction. Common surgical methods for managing abnormal arteries include (14): (1) Vascular hitch procedure (for vessels located above the midline level of the kidney’s lower pole): The fibrous connective tissue on the ectopic vessel’s surface is clamped with 2–3 Hem-o-lok clips and fixed to the perirenal fat sac above the renal hilum. (2) Ectopic blood vessel transposition (for vessels below the midline level of the kidney’s lower pole): The kidney’s lower pole is exposed using an ultrasonic knife, and the ectopic vessel is carefully dissociated from its root, ensuring complete separation from the renal pelvis. After severing the ureter, the ectopic vessel is transposed to the dorsal side of the renal pelvis. If compression is still observed after anastomosis, the ectopic vessel can be fixed to the fascia of the psoas major muscle above the anastomosis to prevent recurrent compression of the anastomosis.

Based on a thorough analysis of the patient’s clinical data, we determined that the severe right hydronephrosis was attributed to a combination of retrocaval ureter and right ureteropelvic junction obstruction, necessitating simultaneous surgical intervention. Considering only a single stenosis cause could lead to its oversight during surgery, thereby failing to eliminate the obstruction comprehensively and adversely impacting the patient’s prognosis.

In a recent case report of retrocaval ureter (15), the surgeon employed traditional open surgery, with imaging data revealing a large incision and a restricted surgical field of view. Traditional open surgery is characterized by extensive trauma, longer incisions, prolonged postoperative recovery, and a higher incidence of complications. In contrast, laparoscopy provides the benefits of smaller incisions, faster postoperative recovery, decreased complications, as well as earlier mobilization and resumption of oral intake. Consequently, the patient underwent laparoscopic retrocaval ureteral realignment combined with right pyeloureteroplasty. During the procedure, ectopic vascular transposition was employed to address the anomaly in the right renal artery. The ureter was carefully dissected, and the red catheter was gently manipulated to safeguard the supporting blood vessels, while avoiding injury to the malformed inferior vena cava and misidentification of the right renal artery, which could potentially result in severe intraoperative complications like massive hemorrhage and partial renal ischemia or necrosis. Post-surgery, the patient exhibited satisfactory recovery.

Three months post-operation, the patient’s right renal hydronephrosis showed substantial improvement compared to the preoperative state. However, due to the prolonged duration of obstruction, morphological changes indicative of hydronephrosis persisted in the resected renal pelvis. Nevertheless, the patient’s renal function recovered remarkably. We will maintain rigorous follow-up and closely monitor any changes in renal pelvis morphology and renal function.

In summary, the coexistence of inferior vena cava and renal artery variations is uncommon in clinical practice, and their combined occurrence leading to hydronephrosis is even more rare. Comprehensive preoperative imaging assessments and meticulous film interpretation are crucial for the successful execution of the surgery. Laparoscopic retrocaval ureteral realignment, coupled with concurrent right pyeloureteroplasty, emerges as a safe, feasible, and effective therapeutic approach for managing this condition.

## Data Availability

The original contributions presented in the study are included in the article/supplementary material, further inquiries can be directed to the corresponding author.
